# The Stringent Response Inhibits DNA Replication Initiation in E. coli by Modulating Supercoiling of *oriC*

**DOI:** 10.1128/mBio.01330-19

**Published:** 2019-07-02

**Authors:** James A. Kraemer, Allen G. Sanderlin, Michael T. Laub

**Affiliations:** a Department of Biology, Massachusetts Institute of Technology, Cambridge, Massachusetts, USA; b Howard Hughes Medical Institute, Massachusetts Institute of Technology, Cambridge, Massachusetts, USA; National Institute of Child Health and Human Development (NICHD); University of Illinois at Urbana Champaign; University of Wisconsin-Madison

**Keywords:** DNA topology, DnaA, ppGpp, replication initiation, stringent response

## Abstract

The stringent response enables bacteria to respond to a variety of environmental stresses, especially various forms of nutrient limitation. During the stringent response, the cell produces large quantities of the nucleotide alarmone ppGpp, which modulates many aspects of cell physiology, including reprogramming transcription, blocking protein translation, and inhibiting new rounds of DNA replication. The mechanism by which ppGpp inhibits DNA replication initiation in Escherichia coli remains unclear. Prior work suggested that ppGpp blocks new rounds of replication by inhibiting transcription of the essential initiation factor *dnaA*, but we found that replication is still inhibited by ppGpp in cells ectopically producing DnaA. Instead, we provide evidence that a global reduction of transcription by ppGpp prevents replication initiation by modulating the supercoiling state of the origin of replication, *oriC*. Active transcription normally introduces negative supercoils into *oriC* to help promote replication initiation, so the accumulation of ppGpp reduces initiation potential at *oriC* by reducing transcription. We find that maintaining transcription near *oriC*, either by expressing a ppGpp-blind RNA polymerase mutant or by inducing transcription from a ppGpp-insensitive promoter, can strongly bypass the inhibition of replication by ppGpp. Additionally, we show that increasing global negative supercoiling by inhibiting topoisomerase I or by deleting the nucleoid-associated protein gene *seqA* also relieves inhibition. We propose a model, potentially conserved across proteobacteria, in which ppGpp indirectly creates an unfavorable energy landscape for initiation by limiting the introduction of negative supercoils into *oriC*.

## Abstract (precis)

To survive bouts of starvation, cells must inhibit DNA replication. In bacteria, starvation triggers production of a signaling molecule called ppGpp (guanosine tetraphosphate) that helps reprogram cellular physiology, including inhibiting new rounds of DNA replication. While ppGpp has been known to block replication initiation in Escherichia coli for decades, the mechanism responsible was unknown. Early work suggested that ppGpp drives a decrease in levels of the replication initiator protein DnaA. However, we found that this decrease is not necessary to block replication initiation. Instead, we demonstrate that ppGpp leads to a change in DNA topology that prevents initiation. ppGpp is known to inhibit bulk transcription, which normally introduces negative supercoils into the chromosome, and negative supercoils near the origin of replication help drive its unwinding, leading to replication initiation. Thus, the accumulation of ppGpp prevents replication initiation by blocking the introduction of initiation-promoting negative supercoils. This mechanism is likely conserved throughout proteobacteria.

## INTRODUCTION

All organisms must coordinate their growth with DNA replication. In particular, when nutrients become scarce, cells must arrest DNA replication. A failure to arrest replication can have deleterious consequences, including genome instability and a loss of cell viability. Bacteria, which often experience highly variable environmental conditions, including periods of nutrient starvation, have evolved elaborate survival responses that include the ability to downregulate DNA replication (1–3). However, the molecular mechanisms that control DNA replication following nutrient limitation remain poorly understood.

For most bacteria, including Escherichia coli, many nutrient limitations induce the so-called “stringent response” (reviewed in reference 3), during which the cell rapidly produces millimolar quantities of the small-molecule messengers pppGpp and ppGpp (referred to here as ppGpp for simplicity), which reprogram cell physiology to a slow-growing state, including arresting the initiation of new rounds of DNA replication. ppGpp is produced during amino acid starvation by the ribosome-associated synthetase RelA, which senses the presence of uncharged tRNAs in the A-site (3, 4). In E. coli, ppGpp binds directly to two sites on RNA polymerase (RNAP) (5, 6) to downregulate the transcription of genes required for rapid growth, especially the seven rRNA loci (7), while activating the transcription of some genes related to stress tolerance and amino acid synthesis (8–11). ppGpp also attenuates other cellular processes, such as translation and nucleotide synthesis, by binding directly to a range of proteins (3, 12–15). A recent study using capture-compound mass spectrometry identified ∼50 candidate proteins that are directly bound by ppGpp (15). Notably, however, no proteins involved in replication initiation were identified.

**FIG 1:**
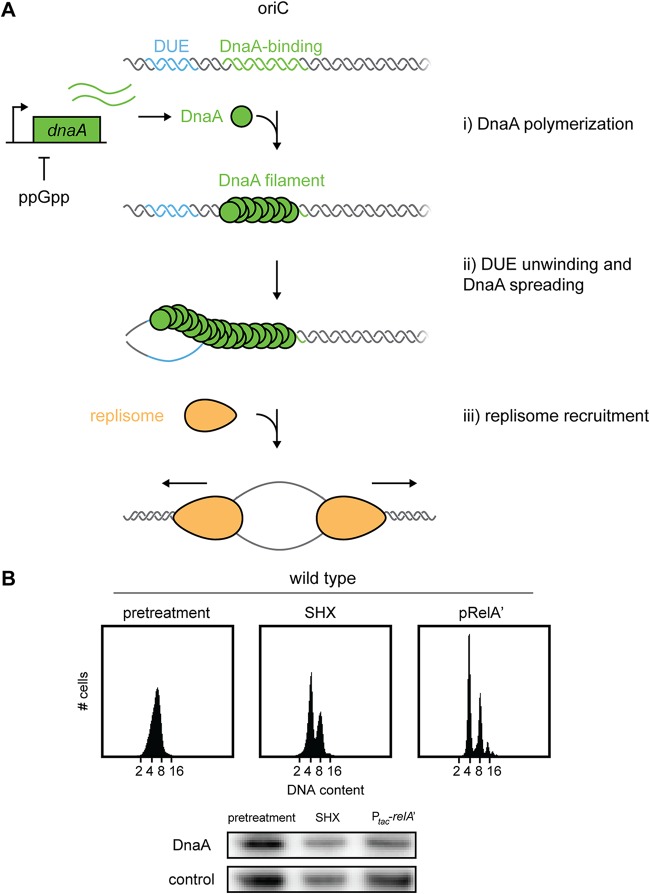
ppGpp inhibits DNA replication initiation in E. coli. (A) Schematic of replication initiation in E. coli and the prevailing model for inhibition by ppGpp. There are three major steps to initiation. (i) DnaA (green circles) assembles onto the double-stranded DnaA-binding region (green). (ii) The DnaA filament spreads onto the melted, single-stranded DNA unwinding element (DUE [cyan]). (iii) DnaA recruits components of the replisome (orange) to initiate DNA replication. (B) Representative flow cytometry profiles of wild-type E. coli grown in LB. Cells were grown to an OD_600_ of ∼0.2, treated as follows, and then fixed for analysis: pretreatment (left panel), addition of 1 mg/ml serine hydroxamate for 90 min (middle panel), induction of RelA′ with 100 μM IPTG for 90 min (right panel). (Bottom) Immunoblot for DnaA in cultures corresponding to flow cytometry profiles in panel A. The control is a nonspecific band seen with the DnaA antibody.

In E. coli, DNA replication initiates from a single origin of replication, *oriC*, and continues bidirectionally to completion at the terminus, *ter*. The timing of replication is controlled in large part by the conserved initiation factor DnaA (16) (Fig. 1A). DnaA is an AAA+ (ATPases associated with diverse cellular activities) protein that binds to 12 consensus sites in *oriC* to then drive melting of the origin, within the DNA unwinding element (DUE), and recruitment of DNA helicase and the helicase loader (17–19). ATP-bound DnaA is required for initiation, and the replication fork stimulates DnaA-ATP hydrolysis after initiation to prevent subsequent reinitiations (16, 17, 20). Newly duplicated *oriC* DNA is hemimethylated and is bound, or sequestered, by SeqA, which occludes DnaA and prevents aberrant reinitiation events (21–23). Eventually, after methylation of *oriC* by the Dam methylase and the regeneration of ATP-DnaA, new rounds of replication can initiate (24, 25). The frequency of replication initiation is tuned to nutrient conditions and growth rate. In the case of starvation, when the stringent response is activated, E. coli cells can finish ongoing rounds of DNA replication, but are inhibited from initiating new rounds of replication (26, 27).

Although ppGpp has been known for decades to inhibit replication initiation in E. coli, the mechanism responsible remains unclear (26, 28). Previous studies found that ppGpp represses the transcription of *dnaA* and proposed that new rounds of replication are inhibited by a decrease in DnaA levels (29) (Fig. 1A). However, it remains untested whether a ppGpp-driven decrease in DnaA is necessary to arrest replication during the stringent response. Moreover, replication initiation could be controlled by other, DnaA-independent mechanisms. For instance, mutations in *seqA* or *dam* prevent an arrest of DNA replication initiation during the stringent response, but how, or if, SeqA responds to ppGpp is not known (26).

The transcription of genes near *oriC* may also impact DNA replication. Active transcription, but not protein production *per se*, is necessary for new rounds of DNA replication to initiate in E. coli, but the underlying mechanism is unknown (30). One model posits that it is not the transcripts themselves that promote initiation, but rather the supercoils induced by RNAP that promote origin melting (31, 32). Unwinding of the DNA duplex by translocating RNAP necessarily introduces positive supercoils in front and negative supercoils behind it (33). Negative superhelicity is associated with underwinding of the DNA, which would promote origin melting by destabilizing duplex interactions and facilitating strand unwinding. Consistent with this model, the transcription of *mioC*, which reads into *oriC*, inhibits replication initiation, as positively supercoiled DNA is more difficult to melt, whereas the transcription of *gidA*, which reads out away from *oriC*, promotes it (34). *gidA* and *mioC* were suggested to be under stringent control, and transcription of these genes influences the replication of *oriC*-based plasmids (32, 35–37). However, blocking the transcription of *gidA* or *mioC* on the chromosome does not affect replication during growth in the exponential phase (37–39). Thus, the net effect of inhibiting transcription proximal to *oriC* on initiation remains unclear.

Here, we directly tested a model in which ppGpp inhibits new rounds of DNA replication initiation in E. coli by modulating supercoiling and *oriC* topology. We found that, contrary to the prevailing model, a decrease in DnaA levels is not responsible for the arrest of DNA replication by ppGpp and instead that high ppGpp levels indirectly prevent the binding of DnaA to *oriC*. Notably, a strain in which RNAP is insensitive to ppGpp continues to initiate DNA replication following the accumulation of ppGpp. We suggest that ppGpp binding to RNAP promotes replication arrest by downregulating bulk transcription, thereby limiting the introduction of initiation-promoting, negative supercoils into *oriC*. We show that driving transcription near *oriC* with a ppGpp-insensitive T7 promoter can largely suppress the inhibition of replication by ppGpp. Additionally, we find that increasing global negative supercoiling by deleting *seqA* or by inhibiting topoisomerase I (topo I) also allows for continued DNA replication following ppGpp accumulation. Taken all together, our results suggest a new model for the inhibition of DNA replication during the stringent response whereby ppGpp decreases bulk transcription to limit the introduction of negative supercoils into *oriC*, effectively increasing the energy barrier for initiation.

## RESULTS

### Induction of ppGpp leads to DNA replication initiation arrest in E. coli.

To investigate the effects of ppGpp on DNA replication, we used flow cytometry to monitor the DNA content of individual cells. E. coli cells grown to the exponential phase in a rich medium (LB) exhibited a normal distribution of DNA content (Fig. 1B, left), reflecting the asynchronous state of the population with respect to replication. Induction of the stringent response by addition of the serine analogue serine hydroxamate (SHX) for 90 min produced two peaks in the flow cytometry profiles, corresponding to cells with integer chromosome content (Fig. 1B, middle), indicating that most cells completed ongoing rounds of replication and could not initiate anew. Expression of a constitutively active form of the ppGpp synthetase RelA (here called RelA′) from an isopropyl-β-d-thiogalactopyranoside (IPTG)-inducible plasmid, pRelA′ (40), for 90 min led to an even stronger arrest of replication initiation, with almost no cells containing noninteger chromosome content. In subsequent experiments, we primarily used RelA′ induction to produce ppGpp to help ensure that any effects on DNA replication observed were due specifically to ppGpp.

To assess how quickly ppGpp inhibits DNA replication initiation, we repeated the flow cytometry, inducing ppGpp via RelA′ for different periods of time before adding rifampin and cephalexin for 3 h. These two antibiotics block new rounds of replication and cell division, respectively. If ppGpp immediately blocks DNA replication initiation, there should be a progressive decrease in the number of chromosomes per cell as cells in the population can divide (prior to addition of cephalexin), but not initiate new rounds of replication. In contrast, if the block to replication by ppGpp is delayed, there should be a maintenance of or increase in the number of chromosomes per cell. We saw a clear decrease in chromosomes per cell (see Fig. S1A in the supplemental material), supporting the notion that ppGpp acts rapidly to inhibit DNA replication initiation.

10.1128/mBio.01330-19.1 FIG S1 ppGpp immediately inhibits new rounds of DNA replication initiation. Shown are representative time-course run-out flow cytometry profiles for WT cells (A) or cells overexpressing DnaA (B). Cells were grown in LB to an OD_600_ of 0.1, and 0.2% arabinose was added to induce *dnaA* expression for 30 min. RelA′ was induced with 100 μM IPTG, and 1-ml samples were taken and treated with rifampin (rif [300 μg/ml]) and cephalexin (ceph [12 μg/ml]) for 4 h. (C) Representative flow cytometry profile of a synchronized culture of Caulobacter crescentus indicating signal for one ∼4-Mbp chromosome. Download FIG S1, PDF file, 0.1 MB. Copyright © 2019 Kraemer et al. 2019 Kraemer et al. This content is distributed under the terms of the Creative Commons Attribution 4.0 International license.

### A reduction in DnaA levels is not the cause of ppGpp-induced replication arrest.

**FIG 2:**
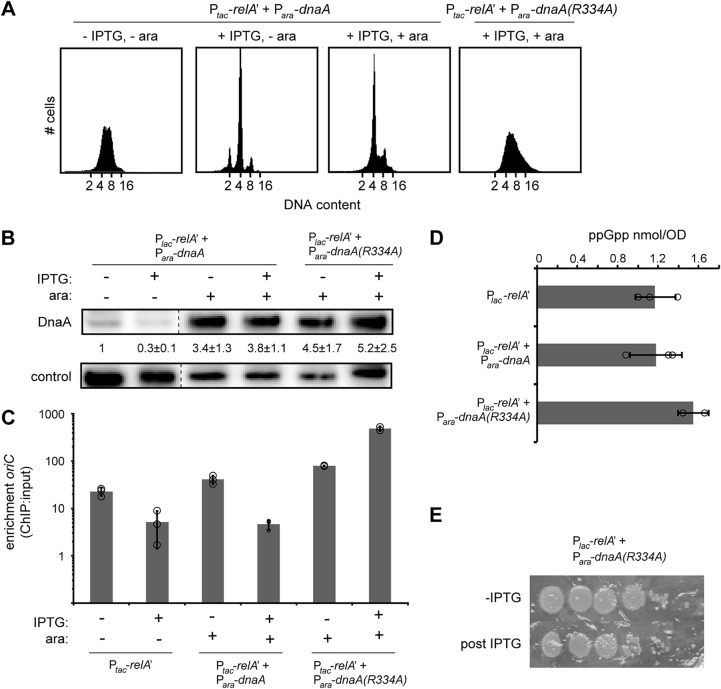
A decrease in DnaA protein levels is not responsible for the inhibition of replication initiation by ppGpp. (A) Representative flow cytometry analysis of cells harboring P*_tac_*-*relA'* and P*_ara_*-*dnaA* or P*_ara_*-*dnaA*(*R334A*) grown in LB. Overnight cultures were diluted back to an OD_600_ of 0.01 in fresh media, grown to an OD_600_ of ∼0.1, treated as follows, and then fixed for analysis: pretreatment (left), RelA′ induction with 100 μM IPTG for 90 min (second from left), DnaA [or DnaA(R334A)] induction with 0.2% arabinose for 30 min followed by RelA′ induction with 100 μM IPTG for 90 min [pDnaA, second from right; pDnaA(R334A), right]. (B) Immunoblots for DnaA in LB for untreated cells and for cells induced for RelA′ and/or DnaA or DnaA(R334A). DnaA levels were normalized to pretreatment levels and quantified from three independent blots. Control is a nonspecific band seen with the DnaA antibody. (C) DnaA association with *oriC* assayed by ChIP-qPCR enrichment. Relative amount of *oriC* DNA in a DnaA immunoprecipitate compared to input was quantified by qPCR. RelA′ was induced by addition of 1 mM IPTG for 30 min prior to fixation. DnaA and DnaA(R334A) were induced by addition of 0.2% arabinose for 30 min. *oriC* DNA levels were quantified using primers against *oriC* and normalized to a locus near the terminus (*relB*) that DnaA does not bind. Enrichment was calculated as the ratio of normalized *oriC* in the ChIP to the input DNA. Error bars represent standard deviation of enrichment from three replicates. (D) Quantification of ppGpp levels in wild-type cells harboring pRelA′, pDnaA and pRelA′, or pDnaA(R334A) and pRelA′. Cells were grown in M9-glycerol plus Casamino Acids, and RelA′ was induced with 100 μM IPTG for 30 min. For pDnaA- and pDnaA(R334A)-containing cells, 0.2% arabinose was added for 30 min to induce *dnaA* expression prior to addition of IPTG. Error bars are the standard deviation from three replicates. (E) Representative serial dilution plating of E. coli strains carrying pRelA′ and pDnaA(R334A). Cells were grown to an OD_600_ of ∼0.1, and a sample was taken for serial dilution (top row). DnaA(R334A) expression was induced with 0.2% arabinose for 30 min followed by RelA′ induction with 100 μM IPTG for 90 min, and a second sample was taken for plating (bottom row).

For almost 30 years, the prevailing model for how ppGpp blocks DNA replication initiation has been that ppGpp inhibits new transcription of *dnaA*, leading to a reduction in DnaA levels below that needed for initiation to occur (29) (Fig. 1B). Consistent with this model, our immunoblotting indicated that DnaA levels decreased ∼3-fold upon induction of RelA′ (Fig. 1B). To test if a reduction in DnaA following ppGpp accumulation drives replication arrest, we generated a strain containing the pRelA′ plasmid in which *dnaA* is expressed by an arabinose-inducible promoter on a separate plasmid. When grown in LB without arabinose or IPTG, this strain displayed a distribution of DNA content comparable to the wild type (WT) (Fig. 2A). Addition of IPTG to induce RelA′ for 90 min without arabinose led to an accumulation of cells with integer chromosome content (Fig. 2A), as with cells harboring only the RelA′ plasmid (Fig. 1B). Inducing the expression of DnaA with arabinose for 30 min prior to RelA′ induction for an additional 90 min also led to an accumulation of cells with integer chromosome content (Fig. 2A). Immunoblotting confirmed that DnaA was produced under these conditions (Fig. 2B). In fact, the levels of DnaA were substantially higher than those of cells without arabinose in which DNA replication is ongoing. These results strongly suggest that the decrease in DnaA that occurs in wild-type cells following ppGpp accumulation cannot be solely responsible for inhibition of DNA replication initiation. Instead, ppGpp must either inactivate DnaA or block replication independently of DnaA. We reasoned it unlikely that ppGpp directly inhibits DnaA, as ppGpp has not been found to bind to DnaA or any of its regulators (15).

We also assessed the kinetics of ppGpp-driven inhibition of replication initiation in cells overexpressing DnaA (Fig. S1B). Prior to RelA′ induction, most cells harbored 8 or 16 chromosomes (Fig. S1B). With RelA′ induction for various periods of time (up to 90 min), followed by rifampin and cephalexin treatment, the number of chromosomes per cell progressively decreased. As described above (Fig. S1A), this finding indicates that ppGpp must be rapidly inhibiting DNA replication initiation.

One possibility is that ppGpp prevents DnaA from binding to *oriC*. To test this possibility, we performed chromatin immunoprecipitation followed by quantitative PCR (ChIP-qPCR). As expected during growth in LB, DnaA in wild-type cells robustly associated with *oriC* (Fig. 2C), with >20-fold ChIP enrichment. Following RelA′ expression, the ChIP enrichment of DnaA at *oriC* decreased to ∼5-fold (Fig. 2C). Notably, inducing RelA′ in a strain overexpressing *dnaA* also led to a substantial reduction in the amount of DnaA bound to *oriC*, with enrichment levels comparable to that seen in cells producing RelA′ alone (Fig. 2C). These results indicate that ppGpp somehow decreases the association of DnaA with *oriC*. However, we cannot assess whether this decrease stems from a decrease in DnaA binding to double-stranded DnaA binding sites or to single-stranded DNA formed in the DUE following origin melting (Fig. 1A), or both (also see Discussion).

The inhibition of DNA replication following ppGpp accumulation could indicate that ppGpp affects the ATP-binding status of DnaA. To test this possibility, we expressed a hyperactive variant of DnaA that is deficient for ATP hydrolysis, DnaA(R334A) (41), for 30 min prior to inducing RelA′. This strain no longer exhibited replication arrest following ppGpp accumulation (Fig. 2A), and DnaA was highly enriched at *oriC* in this strain, both before and after induction of RelA′ (Fig. 2C). Notably, DnaA(R334A) expression did not affect ppGpp production, as all strains produced similar amounts of ppGpp following RelA′ induction (Fig. 2D). We observed only a modest loss of plating efficiency before and after inducing DnaA(R334A) and RelA′ (Fig. 2E), indicating that DnaA(R334A) expression was generally not lethal on the time scale of our experiments. These results could indicate that ppGpp blocks replication by inhibiting DnaA activity, possibly by stimulating its ATPase activity. Alternatively, ppGpp may block replication independent of DnaA, and the oversupply of hyperactive DnaA may simply be sufficient to overcome the inhibition of this parallel, ppGpp-dependent mechanism of replication control.

### ppGpp binding to RNA polymerase contributes to DNA replication arrest.

**FIG 3:**
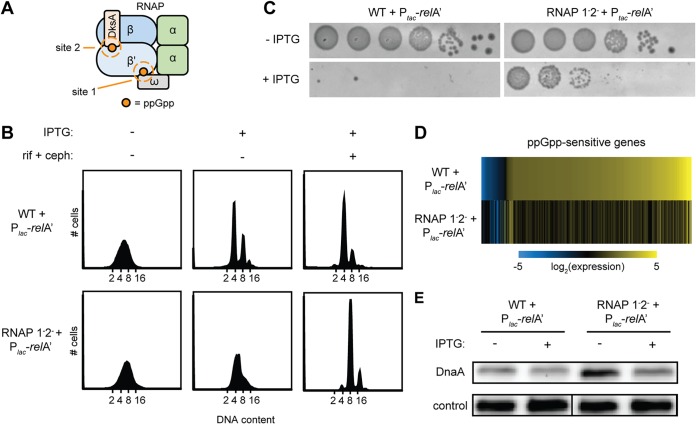
RNAP mutants with decreased ppGpp-binding affinity continue DNA replication upon ppGpp induction. (A) Schematic of RNAP showing the ppGpp-binding sites (orange) mutated in the RNAP 1^−^2^−^ strain. (B) Representative flow cytometry profiles of WT (top row) and RNAP 1^−^2^−^ (bottom row) cells, each harboring the pRelA′ plasmid grown in M9GAV medium, treated as follows, and then fixed for analysis: pretreatment (left panel), RelA′ induction for 90 min by the addition of IPTG (50 μM to WT, 150 μM to RNAP 1^−^2^−^) (middle panel), and addition of rifampin (300 μg/ml) and cephalexin (12 μg/ml) to IPTG-treated cells for 4 h (right panel). (C) Serial dilution plating of WT and RNAP 1^−^2^−^ cells, each harboring the pRelA′ plasmid, on M9GAV agar plates containing 0, 50 μM (WT), or 150 μM (RNAP 1^−^2^−^) IPTG and 100 μg/μl carbenicillin. Cells were grown to an OD_600_ of 0.2, and dilutions of 10^−1^ to 10^−6^ were plated. Plates were incubated for ∼24 h at 37°C. (D) RNAP 1^−^2^−^ cells are transcriptionally blind to ppGpp induction. A heat map shows log_2_ fold changes for genes with expression changing >2 log_2_ in the WT or RNAP 1^−^2^−^ strain 15 min following RelA′ induction with 50 μM (WT) or 150 μM (RNAP 1^−^2^−^) IPTG. Fold changes were calculated as a ratio of reads per kilobase per million (RPKM) between RNA harvested from samples collected 15 min after addition of IPTG and RNA from samples collected immediately prior to IPTG addition. (E) Representative immunoblot for DnaA in WT and RNAP 1^−^2^−^ cells with and without the induction of RelA′ for 90 min with 50 μM (WT) or 150 μM (RNAP 1^−^2^−^) IPTG. The control is a nonspecific band seen with the DnaA antibody.

ppGpp could affect DNA replication either by binding directly to a protein involved in replication initiation or by modulating the transcription of genes involved in replication. We favored the latter possibility, as a recent study examining direct ppGpp targets did not yield any proteins directly implicated in DNA replication initiation (15). To test whether ppGpp-dependent control of replication initiation involves its ability to bind and reprogram RNA polymerase (RNAP), we used a strain, referred to here as the RNAP 1^−^2^−^ strain, in which several mutations in *rpoC* and *rpoZ* effectively eliminate ppGpp binding to RNAP (Fig. 3A) (5). We introduced the pRelA′ plasmid into the RNAP 1^−^2^−^ strain and grew the resulting strain and a wild-type control also harboring pRelA′ to the exponential phase in M9GAV medium supplemented with nucleobases. This medium was previously found to support rapid and comparable growth of the wild type and the RNAP 1^−^2^−^ strain (15). For the control strain, ppGpp production led to a nearly complete arrest of DNA replication (Fig. 3B), as before (Fig. 1B). In clear contrast, DNA replication continued in the RNAP 1^−^2^−^ strain expressing RelA′, with no peaks in flow cytometry corresponding to cells with integer chromosome content (Fig. 3B). To ensure that this pattern arose from cells continuing to replicate rather than simply arresting during replication elongation, we treated these cells with rifampin and cephalexin to inhibit replication initiation and cell division, respectively. These cells then accumulated integer chromosome content, indicating that they were, in fact, competent for and engaged in replication elongation (Fig. 3B).

Notably, we found that the plating viability of cells expressing RelA′ improved nearly 1,000-fold in the RNAP 1^−^2^−^ background compared to a wild-type background (Fig. 3C). Thus, although the expression of RelA′ initially blocks the growth of both wild-type and RNAP 1^−^2^−^ strains in shaking cultures (15), the RNAP 1^−^2^−^ strain can eventually form colonies with much higher efficiency. This result implies that the inhibition of DNA replication is normally a key facet of growth control by ppGpp that is substantially reduced in the RNAP 1^−^2^−^ strain, likely due to the direct transcriptional effects of ppGpp.

To confirm that the transcriptional response to ppGpp is, in fact, attenuated in the RNAP 1^−^2^−^ strain, we performed transcriptome sequencing (RNA-seq) on the wild-type and RNAP 1^−^2^−^ strains harboring pRelA′ grown in M9GAV to mid-exponential phase. RNA was extracted and sequenced 0 and 15 min after inducing RelA′. Consistent with prior studies (8, 9), dramatic changes to the transcriptome were observed in the wild-type strain following ppGpp production, with 329 genes exhibiting >4-fold changes in expression after 15 min (Fig. 3D). In sharp contrast, for the RNAP 1^−^2^−^ strain, only 25 genes exhibited a >4-fold change in expression after 15 min, and several of these genes (e.g., *relA* and *lacZYA*), are associated with the addition of IPTG used to induce expression of RelA′. These findings confirm that the RNAP 1^−^2^−^ strain does not respond to ppGpp by mounting a canonical stringent response gene expression program.

Our results indicated that a block in DNA replication initiation by ppGpp requires its binding to RNA polymerase. This finding could indicate that ppGpp-bound RNA polymerase induces the expression of a replication inhibitor or represses expression of an essential replication component. However, inspection of the list of genes induced and repressed most significantly by ppGpp did not reveal any obvious candidates. Thus, we considered an alternative explanation for how ppGpp controls DNA replication via RNA polymerase. As noted earlier, active transcription, particularly near *oriC*, may help promote replication initiation by introducing or maintaining negative superhelicity near the origin, helping to melt the DNA duplex. In addition to modulating the transcription of many mRNAs (8, 9, 42), ppGpp represses transcription from rRNA promoters (7), which normally account for >80% of transcription in E. coli and are clustered near the origin, with the closest, *rrnC*, only 12 kb from *oriC* (43). Thus, the inhibition of transcription by ppGpp during the stringent response could, in principle, diminish the introduction of negative supercoils near *oriC*, making the origin more difficult to melt, leading to the observed inhibition of replication initiation.

### Driving transcription near *oriC* allows for continued replication in the presence of ppGpp.

**FIG 4:**
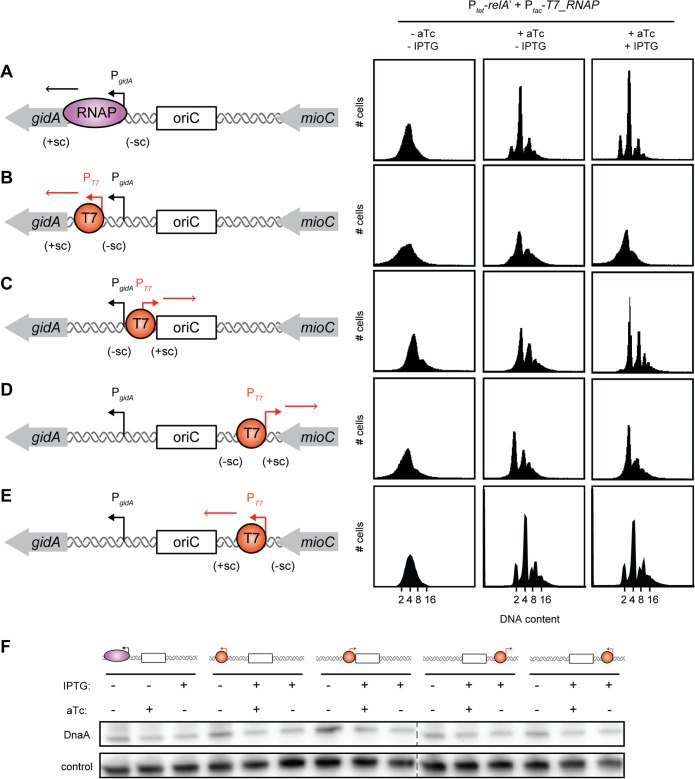
Continued replication in cells with constitutive transcription by T7 RNAP next to *oriC*. (Left panels) (A) Schematic of the *oriC* region showing the genes *mioC* and *gidA* as well as RNAP (purple) for WT. Transcription of *gidA* (black arrow) by RNAP normally introduces negative supercoils (−sc) into *oriC*. (B to E) Schematic of *oriC* showing the location and direction of the engineered T7 promoter with transcript produced by T7 RNAP shown as an orange arrow. (Right panels) Flow cytometry profiles of strains from panels A to E grown in M9-glycerol plus Casamino Acids. IPTG (1 mM) was added to induce T7 RNAP, and 100 nM aTc was added to induce RelA′ for 90 min. Only cells with a T7 promoter facing away from *oriC* continue replication in the presence of ppGpp. (F) Representative immunoblot for DnaA in all strains tested above with or without 1 mM IPTG and 100 nM aTc to induce T7 RNAP and RelA′ as in panels A to E. The control is a nonspecific band in the DnaA antibody.

To test the hypothesis that a global decrease in transcription, particularly of rRNA loci, by ppGpp increases the energy barrier for replication initiation, we sought to examine whether inducing transcription near *oriC*, in a ppGpp-independent manner, would allow replication to continue even after the accumulation of ppGpp. To this end, we introduced a T7 RNAP-dependent promoter (P_T7_) on either side of *oriC*, within the *gidA* promoter region or between *oriC* and the end of the *mioC* coding region (Fig. 4). In each location, the T7 promoter was inserted in either of two orientations, with transcription reading into or away from *oriC* (Fig. 4). Importantly, transcription from P_T7_ is insensitive to ppGpp, as T7 RNAP lacks the ppGpp-binding sites found on E. coli RNAP. For each of the four strains harboring P_T7_ and the wild-type control, we introduced plasmids carrying P*_tac_*-*T7_RNAP* and P*_tet_*-*relA*′ to enable inducible expression of T7 RNAP and RelA′, respectively. Induction of RelA′ in wild-type control cells caused a robust arrest of DNA replication (Fig. 4A). As expected, the expression of T7 RNAP in these cells (which lack a T7-inducible promoter) prior to the expression of RelA′ did not affect the ability of ppGpp to induce an arrest of replication initiation (Fig. 4A).

In stark contrast to the control strain, cells harboring P_T7_ within the *gidA* promoter and reading away from the origin (Fig. 4B), which should introduce negative supercoils into *oriC*, did not show a complete arrest of DNA replication when T7 RNAP was induced for 2 h prior to RelA′ induction (Fig. 4B, right panels). We observed a significant decrease in the number of cells with integer chromosome content for this strain even prior to T7 RNAP induction, likely due to leaky expression from the P*_tac_* promoter. Similar, though weaker, effects were seen when P_T7_ was inserted on the other side of *oriC*, reading away from *oriC* and into *mioC* (Fig. 4D). For the two strains in which P_T7_ was oriented to read into the origin of replication, the induction of T7 RNAP no longer relieved replication arrest following RelA′ expression (Fig. 4C and E). None of these orientations of T7 RNAP transcription resulted in different levels of DnaA after RelA′ expression (Fig. 4F). Collectively, these results indicate that transcription reading away from, but not into, *oriC* is sufficient to bypass the replication block normally induced by ppGpp.

The ability of T7 RNAP-induced transcription to promote DNA replication initiation likely does not result from changes in the expression of *gidA* and *mioC*, the genes flanking *oriC*, as neither gene product controls DNA replication. Instead, we favor a model in which T7 RNAP-induced transcription introduces negative superhelicity in the vicinity of *oriC*, which helps to unwind the DNA duplex and promote replication initiation. This interpretation is consistent with the documented effects of *oriC*-proximal transcription on replication initiation *in vitro* (44, 45).

### Replication arrest is relieved by some nucleoid-associated protein mutants.

**FIG 5:**
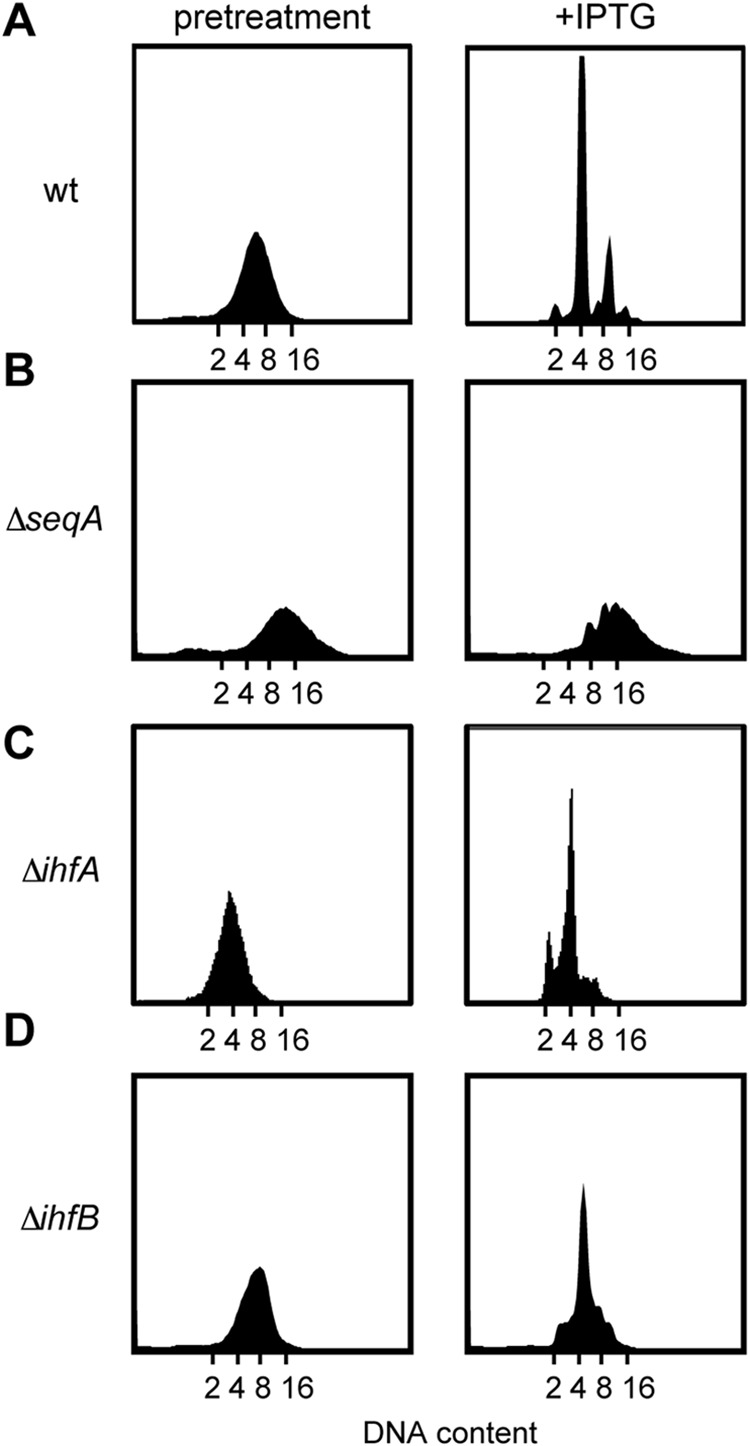
Effects of SeqA and IHF on ppGpp-induced block of DNA replication. Shown are representative flow cytometry profiles of the (A) wild-type or (B) Δ*seqA*, (C) Δ*ihfA*, and (D) Δ*ihfB* strains grown to an OD_600_ of ∼0.2 in LB. Pretreatment values are shown to the left, and results 90 min postinduction of RelA′ with 100 μM IPTG are shown to the right.

In addition to transcription, nucleoid-associated proteins (NAPs) can also have a major effect on supercoiling. Indeed, a Δ*seqA* mutant was previously found to bypass ppGpp-mediated replication arrest (26), which we saw as well (Fig. 5B). The prior work on SeqA indicated that the defects in replication arrest following ppGpp accumulation in the Δ*seqA* strain do not result from an overinitiation of replication in *seqA* cells or from the lack of direct binding of SeqA to *oriC* (26). Additionally, the specificity of SeqA in bypassing replication arrest was not reported. We therefore tested whether null mutations of several other NAPs, including Fis, HU, H-NS, IHF (integration host factor), and Dps, could bypass ppGpp-induced replication arrest. For each mutant strain, except the Δ*ihfA* and Δ*ihfB* mutants, the induction of ppGpp via expression of RelA′ still produced clear peaks in flow cytometry corresponding to integer chromosome content (Fig. 5C and D; see Fig. S2A to F in the supplemental material). Thus, SeqA is relatively specific in affecting replication initiation arrest by ppGpp. The bypass of replication arrest seen with *seqA* and *ihfA*/*ihfB* mutants does not stem from a difference in the accumulation of ppGpp, which is comparable to the wild type for the *seqA* mutant and even slightly higher for the *ihfA* and *ihfB* mutants (Fig. S2G). Similarly, the bypass seen with these mutants is not due to an accumulation of DnaA (Fig. S2H). Instead, we suggest that *seqA* likely affects replication by impacting chromosome topology. To the best of our knowledge, SeqA is the only one of the NAPs tested that introduces positive supercoils into DNA (46–52) and that directly interacts with topoisomerase IV (topo IV) (53), leading to higher negative superhelicity of the chromosome when deleted (54, 55). IHF is not known to affect chromosome superhelicity; however, the bypass in Δ*ihfA* and Δ*ihfB* may result from the fact that IHF normally promotes ATP hydrolysis by DnaA at the *datA* locus (56).

10.1128/mBio.01330-19.2 FIG S2 Effects of nucleoid-associated proteins on ppGpp-induced block of DNA replication. (A to F) Representative flow cytometry profiles of (A) Δ*fis*, (B) Δ*dps*, (C) Δ*hupA*, (D) Δ*hupB*, (E) Δ*hns*, and (F) P_ara_-*hns* strains grown to an OD_600_ of ∼0.2 in LB. Pretreatment (left) and 90 min postinduction of RelA′ with 100 μM IPTG (right). For pH-NS (F), 0.2% arabinose was added to induce *hns* transcription for 60 min prior to fixation or IPTG addition. (G) Quantification of ppGpp levels in mutant strains harboring pRelA′. Cells were grown in M9-glycerol plus Casamino Acids, and RelA′ was induced with 100 μM IPTG for 30 min. For pH-NS-containing cells, 0.2% arabinose was added for 60 min to induce *hns* expression prior to addition of IPTG. Error bars are the standard deviation from three replicates. (H) Immunoblot for DnaA levels preinduction and 90 min after inducing RelA′ with 100 μM IPTG in the mutant strains as described above. Download FIG S2, PDF file, 0.1 MB. Copyright © 2019 Kraemer et al. 2019 Kraemer et al. This content is distributed under the terms of the Creative Commons Attribution 4.0 International license.

### Inhibiting topo I also prevents ppGpp from inhibiting DNA replication initiation.

**FIG 6:**
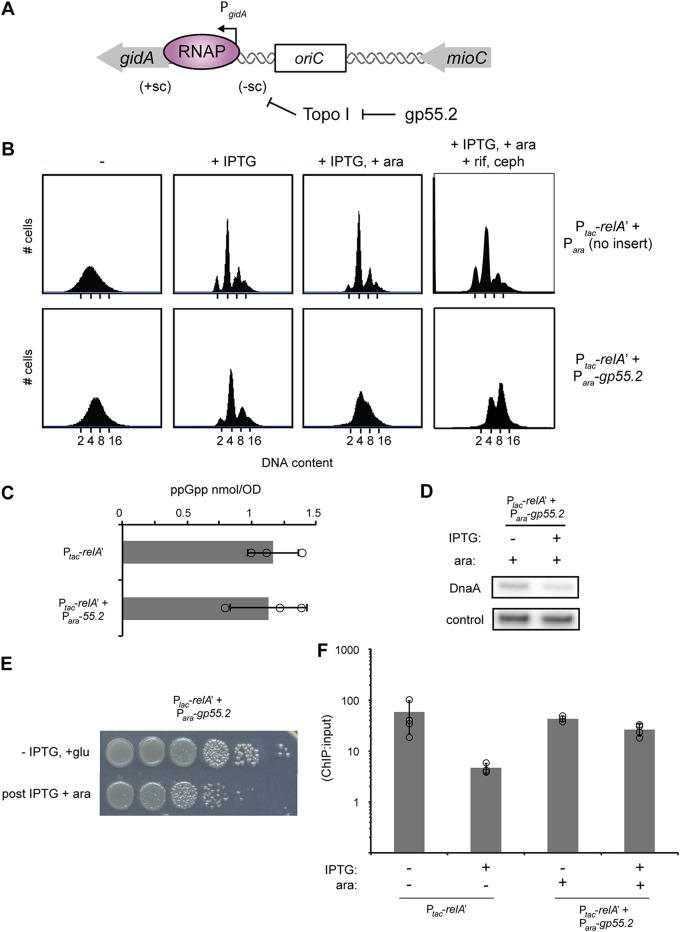
Inhibition of TopA by gp55.2 allows for a bypass of ppGpp-induced replication arrest. (A) Schematic of *oriC* indicating that gp55.2 from T4 phage inhibits topo I from relieving negative supercoils. (B) Flow cytometry profiles for cells harboring pRelA′ and a low-copy-number plasmid with no insert (top) or gp55.2 (bottom) grown in M9-glycerol plus Casamino Acids. Cells were fixed before treatment (left panel), 90 min after RelA′ induction with 100 μM IPTG (second panel from the left), or 15 min after the addition of 0.2% arabinose to induce gp55.2 followed by RelA′ induction with 100 μM IPTG for 90 min (third panel from the left). The latter populations of cells were also treated with rifampin and cephalexin as in Fig. 3B to allow run-out of ongoing rounds of replication (fourth panel from the left). (C) Quantification of ppGpp levels in strains harboring gp55.2 and pRelA′. Cells were grown in M9-glycerol plus Casamino Acids, and 0.2% arabinose was added to induce gp55.2 expression for 15 min, followed by 100 μM IPTG RelA′ for 30 min. Error bars are the standard deviation from three replicates. (D) Immunoblot for DnaA levels in cells expressing gp55.2 before and 90 min after inducing RelA′ with 100 μM IPTG. gp55.2 expression was induced for 15 min by addition of 0.2% arabinose. (E) Serial dilution plating of E. coli carrying pRelA′ and pgp55.2. Cells were grown to an OD_600_ of ∼0.1 without induction and plated on M9 agar plates containing 0.2% glucose to repress gp55.2 expression (top row) or first grown in the presence of 0.2% arabinose (for 105 min) to induce gp55.2 and with 100 μM IPTG for 90 min to induce RelA′ before plating on M9 with 0.2% glucose (bottom row). (F) DnaA association with *oriC* assayed by ChIP-qPCR enrichment. The relative amount of *oriC* DNA in a DnaA immunoprecipitate compared to input was quantified by qPCR. RelA′ was induced by addition of 100 μM IPTG for 30 min prior to fixation. *55.2* expression was induced by addition of 0.2% arabinose for 20 min. *oriC* DNA levels were quantified using primers against *oriC* and normalized to a locus near the terminus (*relB*) that DnaA does not bind. Enrichment was calculated as the ratio of normalized *oriC* in the ChIP to the input DNA. Error bars represent standard deviation of enrichment from three or four replicates.

To more directly test our model that the supercoiling status of *oriC* affects replication initiation control by ppGpp, we examined the role of topoisomerase I (topo I [encoded by *topA*]). In most bacteria, including E. coli, topo I is the main enzyme responsible for relaxing negative supercoils, and *topA* mutant strains with reduced function (e.g., the *topA10* strain), have increased global negative DNA superhelicity (57). Notably, *topA* mutants can suppress the temperature sensitivity of a *dnaA* allele, *dnaA46*, which is deficient in replication initiation (58). To manipulate chromosome superhelicity, we took advantage of a recently described T4 phage protein, gp55.2, which specifically inhibits topo I activity (59). We introduced a low-copy-number plasmid with the T4 gene *55.2* under the control of an arabinose-inducible promoter into a wild-type strain along with the IPTG-inducible pRelA′ plasmid. We also built an empty-vector control strain containing the arabinose-inducible plasmid without gene *55.2*. The two strains were grown to early exponential phase before adding arabinose for 15 min, followed by the addition of IPTG for 90 min to produce ppGpp (Fig. 6A and B). Cells harboring an empty vector showed a clear arrest of DNA replication, as expected, whereas cells producing gp55.2 did not. Cells producing gp55.2 still produced amounts of ppGpp equivalent to those seen with the wild type, while levels of DnaA were actually decreased relative to the wild type (Fig. 6C and D). Little cell death occurred due to expression of *55.2* over this time, as we observed only a small loss in colony formation by cells taken 90 min after RelA′ induction (or 105 min after gp55.2 induction) (Fig. 6E). Thus, it is unlikely that the flow cytometry profile indicating cells with noninteger chromosome content results from a toxicity of gp55.2. Consistent with this conclusion, peaks corresponding to integer chromosome content formed when these cells were treated with rifampin and cephalexin (Fig. 6B). Thus, taken together, these results support a model in which ppGpp normally promotes replication arrest by decreasing negative superhelicity near *oriC*.

Finally, we asked if cells with increased negative supercoiling restored DnaA binding to *oriC* in the presence of ppGpp, as cells displayed continued DNA replication. To this end, we performed ChIP-qPCR on cells expressing gp55.2. The expression of gp55.2 did not increase enrichment of DnaA at *oriC* prior to RelA′ induction (Fig. 6F). However, in contrast to wild-type cells, DnaA was still robustly enriched at *oriC* after 30 min of RelA′ induction (Fig. 6F). This result suggests that increasing negative supercoiling can restore occupancy of DnaA at *oriC* in the presence of ppGpp, supporting a model in which the activity of DnaA at *oriC* is at least partially dependent on negative supercoiling.

## DISCUSSION

### ppGpp inhibits replication initiation in E. coli through its effects on DNA supercoiling.

ppGpp has long been known to inhibit new rounds of DNA replication in E. coli while allowing ongoing rounds of replication to finish. However, the mechanism of this inhibition had been unclear. Previous work suggested that decreased transcription of *dnaA* was responsible (29), but we found that a strain constitutively producing DnaA still exhibited replication arrest following ppGpp induction. This finding could indicate that ppGpp directly inhibits DnaA, or another replication initiation protein, but no candidates were identified in a recent, systematic study of ppGpp binding partners (15).

Instead, the work presented here suggests that ppGpp inhibits replication initiation indirectly by affecting the supercoiling state of the origin of replication. We found several seemingly unrelated genetic perturbations that allowed cells to bypass the inhibition of DNA replication by ppGpp: (i) mutations that eliminate the ppGpp-binding sites on RNAP and thereby remove ppGpp’s direct influence on transcription (Fig. 3), (ii) constitutive transcription from a T7 promoter located near *oriC* and specifically oriented in the same direction as replication (Fig. 4), (iii) deletion of *seqA* (Fig. 5), and (iv) inhibition of topo I by the T4 protein gp55.2 (Fig. 6). A common thread connecting these perturbations is that each increases negative supercoiling at *oriC*, and negative supercoiling promotes replication initiation (31, 44), likely by making duplex DNA easier to melt.

The E. coli chromosome normally has net negative superhelicity, with supercoiling controlled in large part by topoisomerases, particularly DNA gyrase, which relaxes positive supercoils and introduces negative supercoils, and topo I, which relaxes negative supercoils (60). Negatively supercoiled DNA is underwound and, therefore, more prone to melt, thereby promoting replication initiation. Thus, the structure and topology of the chromosome are increasingly appreciated as major factors in the control of replication initiation (31). Indeed, mutations in *topA* (topo I) suppress the lethality of the temperature-sensitive *dnaA46* allele, which is deficient in active DnaA protein at the restrictive temperature (58). Conversely, mutations in the *gyrB* subunit of DNA gyrase exacerbate the lethality of *dnaA46* (61). Consistent with those prior results, we demonstrated that a ppGpp-driven inhibition of replication initiation was effectively bypassed by expressing T4 gp55.2, an inhibitor of topo I (59).

Our model of replication initiation control during the stringent response through changes in the supercoiling status of *oriC* also helps to explain prior results (26), confirmed here, showing that Δ*seqA* cells continue replicating following an induction of ppGpp. SeqA can directly introduce positive supercoils into DNA (46) and Δ*seqA* cells exhibit increased global negative superhelicity of their chromosomes (54, 55). We also found that IHF is required for a complete block to replication initiation following ppGpp induction. IHF does not introduce positive supercoils like SeqA, but it does directly bind *oriC* (62, 63) and so may affect replication initiation rates directly or by affecting DnaA-ATP levels cooperatively with *datA* (64). Finally, our model fits well with a recent study showing that induction of a gene encoding a transmembrane protein up to 1 Mbp from *oriC* inhibited replication initiation, likely by modulating chromosome structure (65).

Changes in superhelicity around *oriC* that promote unwinding of the DUE are the simplest model to explain the disparate mutations that can bypass a ppGpp-driven inhibition of replication. Consistent with our model, studies examining plasmid topology have shown that the level of negative supercoiling decreases when ppGpp levels increase (66, 67). A direct probing of the local supercoiling state of *oriC* will ultimately be necessary to confirm our model. However, current techniques for assaying chromosome supercoiling *in vivo* do not have the resolution to probe a small (∼250-bp) locus and its immediate proximal regions. Most assays of chromosome topology only provide information on the average supercoiling across the genome (27, 68, 69). Recent efforts to use psoralen, which preferentially binds negatively supercoiled DNA, with cross-linking and deep sequencing do not yet offer the sensitivity or resolution to probe the topological state of specific loci like *oriC* (70).

### Interplay between transcription and replication initiation.

One of the major sources of negative supercoils in bacterial chromosomes is transcription, particularly of rRNA, which accounts for >80% of all transcription (71, 72). In the RNAP 1^−^2^−^ strain, ppGpp cannot inhibit RNAP such that transcription continues unabated, including from *oriC*-proximal promoters like *gidA*. Additionally, six of the seven rRNA loci in E. coli are within 300 kb of *oriC* (with *rrnC* just 12 kb away), and each is oriented codirectionally with replication (43); consequently, continued transcription of these loci following ppGpp accumulation in the RNAP 1^−^2^−^ strain will also continue introducing negative supercoils into *oriC*-proximal DNA, promoting replication initiation (Fig. 3). Similarly, we found that forcing transcription near *oriC* with T7 RNAP, which is insensitive to ppGpp, also allowed cells to continue replicating, but only if the T7 promoter was oriented codirectionally with replication such that it would introduce negative supercoils into *oriC* (Fig. 4). Taken all together, these results indicate that ppGpp inhibits new rounds of DNA replication in E. coli primarily by decreasing negative supercoiling, which results from its inhibition of bulk transcription.

Our results underscore the critical contribution of transcription to DNA replication initiation. Transcription is known to be necessary for DNA replication initiation, but precisely why has not been clear. Rifampin, which inhibits transcription, is a potent inhibitor of DNA replication initiation, but this effect is not due to a lack of new transcription of any specific factor (30), and transcripts near *oriC* do not serve as primers for replication initiation (44). *In vitro* studies using plasmids suggested that transcription near *oriC* may stimulate replication initiation by destabilizing the double helix through the formation of stable R-loops or by the introduction of supercoils by RNAP (44, 45). Our *in vivo* studies of chromosomal replication initiation support the latter idea.

Transcription may also play a role in promoting DnaA binding to *oriC* to promote initiation. We found that the occupancy of DnaA at *oriC*, as assessed by ChIP, decreased substantially in response to ppGpp (Fig. 2A). However, the simplest explanation for this decrease is that it reflects a loss in DnaA binding to single-stranded DNA within the DUE (Fig. 1A), which cannot be as easily melted following ppGpp accumulation. DnaA may still bind double-stranded DNA (i.e., some or all of the DnaA boxes within *oriC*), possibly explaining why DnaA ChIP enrichment is still well above background after ppGpp induction. Consistent with this interpretation, prior biochemical studies demonstrated that the initial binding of DnaA to *oriC* is not supercoiling dependent (73).

### Concluding remarks.

Our findings support a model in which high levels of ppGpp lead to a reduction in supercoiling around *oriC*, effectively increasing the energy required for initiation and, in particular, DNA melting. In this way, ppGpp may help tune, or match, replication initiation to growth rate. Indeed, previous studies have shown that compaction of the E. coli chromosome is correlated with growth rate. At high growth rates, when ppGpp levels are lowest, chromosomes are generally condensed, but following acute starvation, the chromosome becomes relaxed, or decondensed, in a ppGpp-dependent manner, likely due to the block in rRNA transcription (26, 74). The tuning of replication initiation rates by ppGpp and its effects on supercoiling are consistent with an energetic model for the regulation of DNA replication proposed by Magnan and Bates (31), wherein supercoiling status, DnaA-ATP levels, and thermal energy combine to modulate replication initiation. Our results suggest that ppGpp may be a key player in modulating replicative capacity, especially as DnaA protein levels remain constant at different growth rates (75, 76).The mechanism of replication control by ppGpp proposed here may be conserved across proteobacteria. As in E. coli, ppGpp inhibits replication initiation in both Caulobacter crescentus (77–79) and Pseudomonas aeruginosa (80). Additionally, the ppGpp-binding sites mapped for E. coli RNAP are conserved among many proteobacteria (5), and most bacteria have rRNA loci relatively close to their origins that are oriented codirectionally with replication, as in E. coli. Thus, ppGpp binding to RNAP in these organisms may lead to similar changes in transcription and supercoiling, as suggested here for E. coli, producing similar effects on replication initiation. The direct inhibition of RNAP by ppGpp is not conserved beyond the proteobacteria (5, 81); for example, RNAPs from Bacillus subtilis and Thermus thermophilus are insensitive to ppGpp *in vitro* (81–83). However, the transcription of rRNA is still inhibited in these Gram-positive organisms, albeit indirectly, by ppGpp. In B. subtilis, ppGpp production leads to a rapid drop in levels of GTP, the starting nucleotide of rRNA transcripts, leading to an inhibition of transcription (84). Thus, it seems likely that ppGpp could be affecting replication initiation via changes in chromosome topology throughout bacteria.

## MATERIALS AND METHODS

### Growth conditions.

Cells were grown at 37°C in LB (10 g/liter NaCl, 10 g/liter tryptone, and 5 g/liter yeast extract), M9 glycerol-Casamino Acids medium (1× M9 salts, 1 mM MgSO_4_, 100 μM CaCl_2_, 0.4% glycerol, 0.2% Casamino Acids), or M9GAV medium (1× M9 salts, 0.4% glucose, 0.25% each of l-serine and l-threonine, 0.0375% each of l-asparagine and l-glutamine, 0.015% each of all 16 other natural amino acids, 0.2 mM nucleobases, and 1× Kao & Michayluk vitamins) and supplemented with antibiotics as noted at the following concentrations (liquid/plate): carbenicillin, 50/100 μg/ml; kanamycin, 30/50 μg/ml; chloramphenicol, 20/30 μg/ml; and spectinomycin, 50/50 μg/ml. IPTG (100 μM), arabinose (0.2%), or anhydrotetracyline (aTc [100 nM]) was added as an inducer of gene expression unless otherwise noted. For M9GAV experiments, RelA′ was induced with 50 μM IPTG (WT) or 150 μM (RNAP 1^−^2^−^) to achieve comparable induction levels (15). Plates contained 1.2% agar. dl-Serine hydroxamate (Sigma) was added to cells at a concentration of 1 mg/ml. For rifampin runouts, rifampin (300 μg/μl) and cephalexin (12 μg/μl) were added for 4 h.

### Strain construction.

All strains used were derivatives of E. coli MG1655 and can be found in Table S1 in the supplemental material, and all primers used can be found in Table S2 in the supplemental material. *fis*, *hupA*, *hupB*, and *hns* deletions were made by P1 transduction from the Keio knockout collection. Disruption of the *seqA*, *ihfA*, *ihfB*, and *dps* genes was created by λ-Red recombination using the FRT::*kan*::FRT or FRT::*cam*::FRT cassette amplified from pKD4 or pKD3 containing homology to the start and end of the gene coding region. T7 promoter insertions were generated by amplifying the *kan* cassette from pKD4 with primers containing the T7 promoter in the proper orientation and homology to the intergenic regions between *oriC* and *gidA* or *mioC* and integrated using λ-Red recombination. The *kan* marker was subsequently removed using pCP20, leaving an FRT scar. The RNAP 1^−^2^−^ and WT control strains were a generous gift from the lab of R. Gourse (University of Wisconsin, Madison).

10.1128/mBio.01330-19.3 TABLE S1 Plasmids used. Download Table S1, XLSX file, 0.01 MB. Copyright © 2019 Kraemer et al. 2019 Kraemer et al. This content is distributed under the terms of the Creative Commons Attribution 4.0 International license.

10.1128/mBio.01330-19.4 TABLE S2 Strains used. Download Table S2, XLSX file, 0.01 MB. Copyright © 2019 Kraemer et al. 2019 Kraemer et al. This content is distributed under the terms of the Creative Commons Attribution 4.0 International license.

10.1128/mBio.01330-19.5 TABLE S3 Primers used. Download Table S3, XLSX file, 0.01 MB. Copyright © 2019 Kraemer et al. 2019 Kraemer et al. This content is distributed under the terms of the Creative Commons Attribution 4.0 International license.

### Plasmid construction.

pALS13 (pRelA′) was a gift from S. Lovett (Brandeis University). For the *dnaA* and *hns* expression plasmids, the *dnaA* and *hns* genes were amplified from the E. coli chromosome and cloned into pBAD33 by restriction cloning using the SacI and HindIII sites. The R334A mutation was introduced by Quikchange site-directed mutagenesis. The T7 expression plasmid pN565 was a gift from C. Voigt (Massachusetts Institute of Technology). The gp55.2 and empty expression vector pDB2114-101 and pBAD101 were gifts from D. Belin (University of Geneva). pKS22b-hSUMO-DnaA was constructed by restriction cloning using the BamHI and HindIII sites on pKS22b-hSUMO.

### Protein expression and purification.

BL21(DE3) cells harboring pKS22b-hSUMO-DnaA were grown in LB at 37°C with shaking to an OD_600_ of ∼0.5. Cultures were cooled, and protein expression was induced with 1 mM IPTG for 18 h at 18°C. DnaA was purified using a modified protocol previously described (85) with the following exceptions: clarified lysate was batch bound to 2 ml of Ni-nitrilotriacetic acid (NTA) resin (Qiagen), the His_7_-SUMO tag was removed overnight by Ulp1, and the resulting protein was run over a Ni-NTA column to remove His_7_-SUMO and other impurities.

### Flow cytometry.

Cells were fixed by addition of 700 μl 95% ethanol to 300 μl of cells. Cells were harvested at 3,400 × *g* for 4 min, resuspended in 1 ml 50 mM sodium citrate containing 5 μg/ml RNase A (Qiagen), and incubated at 50°C for a minimum of 4 h. RNase-treated cells were then diluted 1:10 in 50 mM sodium citrate with 0.5 μl/ml SYTOX green nucleic acid stain (Thermo Fisher) and analyzed on either a BD Accuri C_6_ or a MACSQuant VYB.

### Immunoblotting.

The equivalent of 1 ml of cells at an OD_600_ of 0.1 was pelleted by centrifugation at 21,000 × *g* for 1 min, the medium was aspirated off, and the cell pellets were snap-frozen in liquid N_2_. Cell pellets were resuspended in 100 μl of 1× SDS loading buffer (Biogen), and proteins were separated by SDS-PAGE on AnyKD gels (Biogen). Blots were probed using antibodies against DnaA—either against the C terminus (a gift from J. Kaguni, Michigan State University), at 1:5,000, or full-length, generated against purified full-length DnaA (Covance) at 1:5,000. The rabbit polyclonal antibody generated against full-length DnaA was purified using a DnaA column made with NHS (*N*-hydroxysuccinimide) resin (Thermo) following the manufacturer’s guidelines.

### Quantification of ppGpp.

ppGpp quantification was carried out as previously reported (15). Briefly, cells were grown in M9-glycerol plus Casamino Acids medium at 37°C with shaking to an OD_600_ of 0.2 to 0.3. ppGpp production was inducted for 30 min with 100 μM IPTG, and cells were harvested onto a 0.22-μm-pore hydrophilic polyvinylidene difluoride (PVDF) membrane by vacuum filtration and immediately plunged into ice-cold lysis solvent (40% methanol, 40% acetonitrile, 20% water). Cells were removed from the membrane by brief sonication, and lysates were normalized to an OD_600_ of 1/ml with lysis solvent. ppGpp was quantified by anion-exchange chromatography using a MonoQ 5/50.

### Chromatin immunoprecipitation and enrichment analysis.

Twenty-milliliter cultures were grown in LB to an OD_600_ of ∼0.3, and RelA′ was induced with 1 mM IPTG for 30 min. For strains carrying *dnaA* plasmids, 0.2% arabinose was added 30 min prior to RelA′ induction or fixation. For strains carrying the *55.2* plasmid, 0.2% arabinose was added 20 min prior to RelA′ induction or fixation. Cells were fixed by the addition of 550 μl 37.5% formaldehyde (Sigma) and 10 mM NaPO_4_ at pH 7.6 and incubated for 20 min at room temperature. Subsequent steps were performed as previously described with 90 μl of precleared lysate reserved as input DNA for qPCR analysis (86, 87).

Determination of *oriC* enrichment was conducted by qPCR. Input DNA was diluted 1:100, and ChIP DNA was diluted 1:10 and mixed with primers for either *oriC* or *relB* (control) and 2× qPCR Master Mix (Kapa). qPCR was conducted as described previously. Enrichment of *oriC* was calculated as a ratio of (*oriC* ChIP/*oriC* input) to (*relB* ChIP/*relB* input) to normalize for origin count in each sample.

### RNA extraction and sequencing.

Wild-type or RNAP 1^−^2^−^ cells harboring pALS13 were grown in M9GAV to an OD of ∼0.2. *relA*′ was induced with 50 μM (WT) or 150 μM (RNAP 1^−^2^−^) IPTG. A prior study demonstrated that these concentrations of IPTG produce similar levels of RelA′ and ppGpp (15); the difference in IPTG concentration needed for similar ppGpp levels likely stems from a reduction in transcriptional activation of the *tac* promoter in the RNAP 1^−^2^−^ strain. Three milliliters of culture was removed 0, 5, 10, and 15 min post-IPTG addition, fixed with 300 μl cold stop solution (95% ethanol, 5% phenol, pH 4.3 [Sigma]), and spun for 1 min at 20,000 × *g*, supernatant was aspirated off, and the culture was snap-frozen in liquid N_2_. RNA was extracted by hot TRIzol (Ambion) lysis followed by purification using the Direct-zol RNA MiniPrep kit (Zymo). DNA was removed by 2× addition of 2 μl Turbo DNase (Thermo) and incubation for 20 min at 37°C, and the RNA was concentrated by ethanol precipitation. rRNA was removed through use of the Ribo-Zero kit for Gram-negative bacteria (Illumina). Sequencing libraries were constructed and sequenced on an Illumina Next-Seq at the MIT Bio Micro Center.

Gene expression data analysis was performed using custom scripts in Python 2.7.6. Reads were mapped as previously described (88) to the MG1655 genome (NC_000913.2) with bowtie2 (version 2.1.0) using the arguments: -D 20 -R 3 -N 0 -L 20 -i S,1,0.50 -p 6 -l 40 -X 300.

### Data availability.

RNA-seq data are available in the GEO database under accession no. GSE128606.
