# Supplementary figures and images for: The Stringent Response Inhibits DNA Replication Initiation in E. coli by Modulating Supercoiling of oriC
Source: mBio. 2019 Jul 2;10(4):e01330-19. doi: 10.1128/mBio.01330-19 (PMC6606810; doi:10.1128/mBio.01330-19)

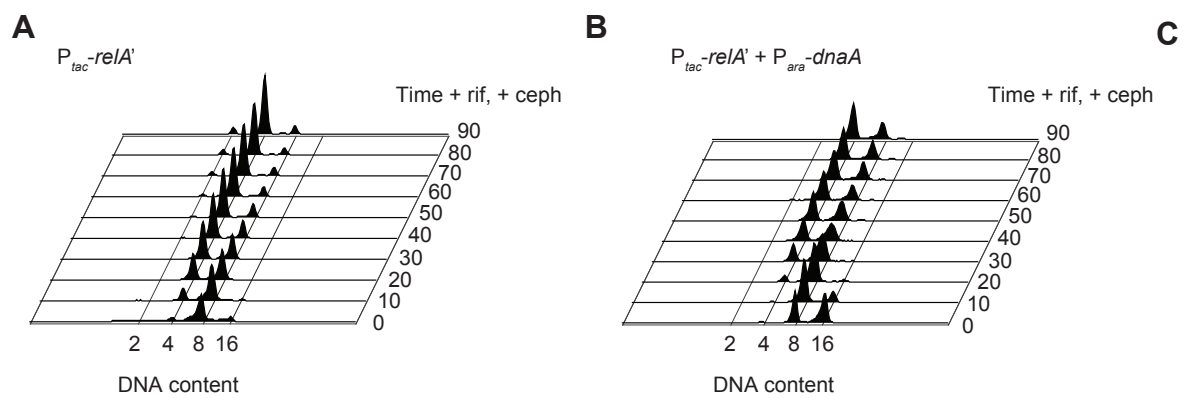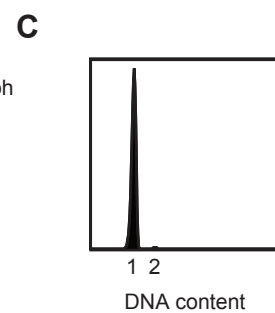

Supplement: FIG S1 [file mBio.01330-19-sf001.pdf]

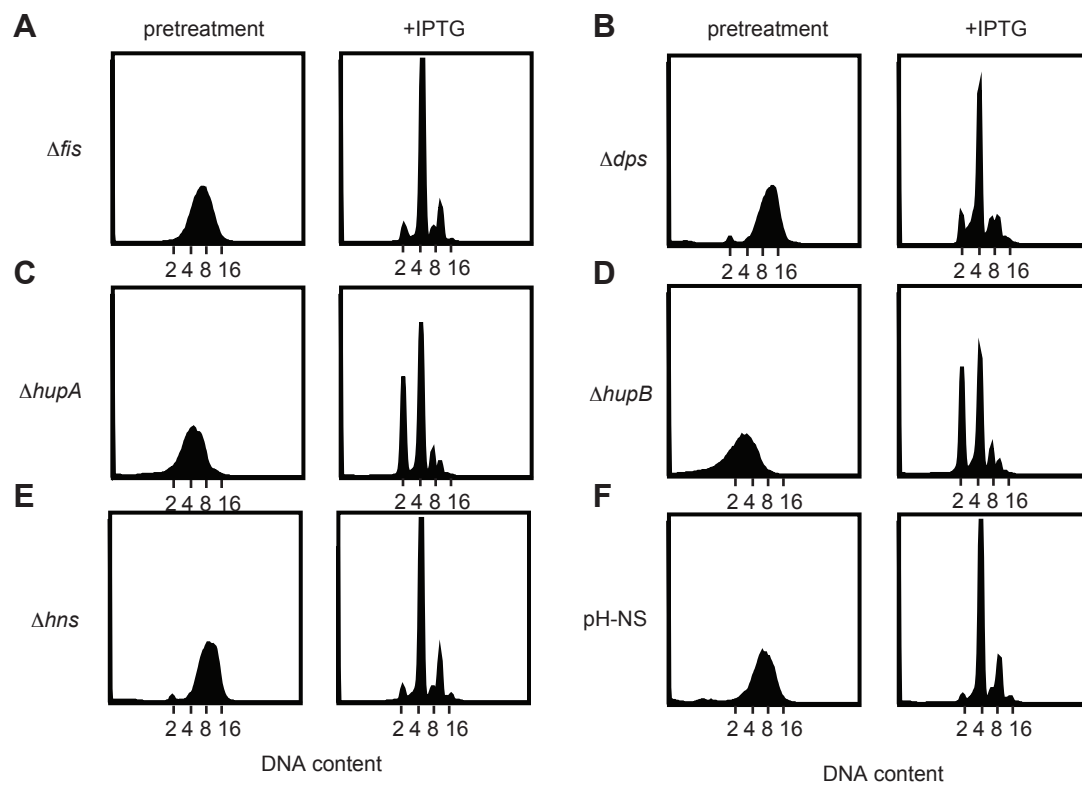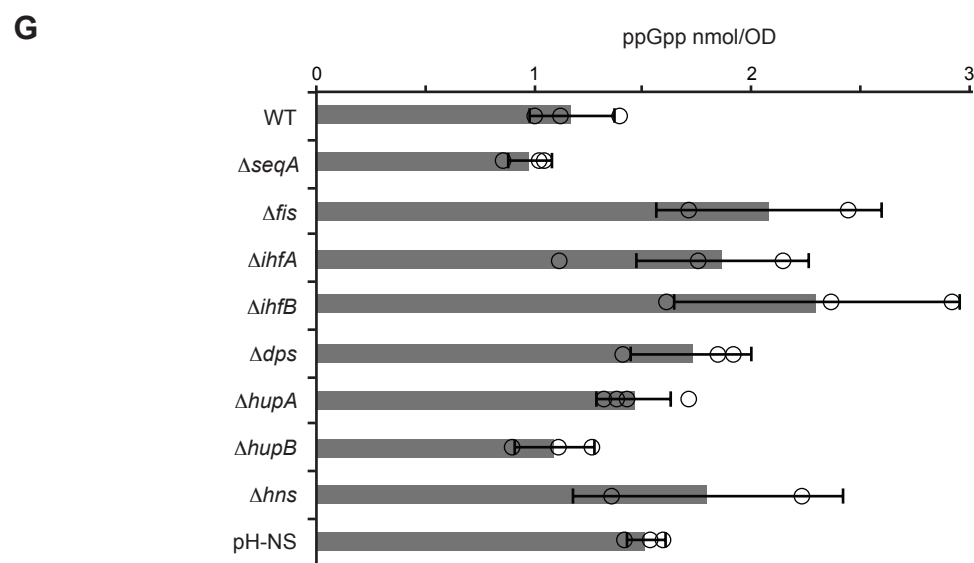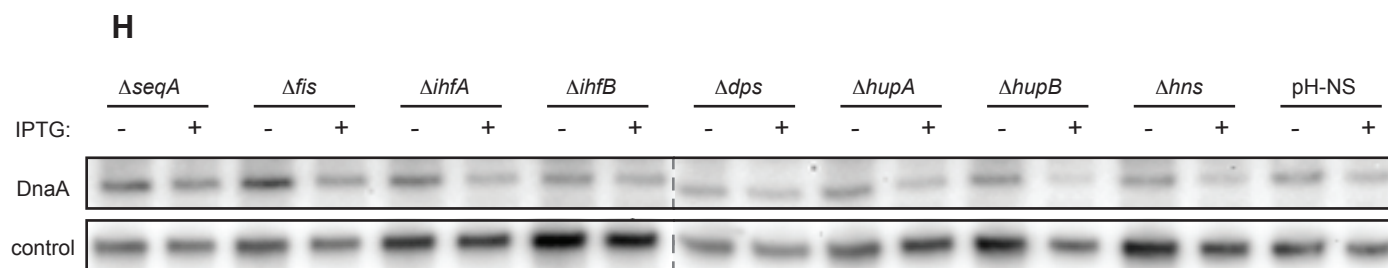

Supplement: FIG S2 [file mBio.01330-19-sf002.pdf]
